# Quercetin Is More Effective than Cromolyn in Blocking Human Mast Cell Cytokine Release and Inhibits Contact Dermatitis and Photosensitivity in Humans

**DOI:** 10.1371/journal.pone.0033805

**Published:** 2012-03-28

**Authors:** Zuyi Weng, Bodi Zhang, Shahrzad Asadi, Nikolaos Sismanopoulos, Alan Butcher, Xueyan Fu, Alexandra Katsarou-Katsari, Christina Antoniou, Theoharis C. Theoharides

**Affiliations:** 1 Molecular Immunopharmacology and Drug Discovery Laboratory, Department of Molecular Physiology and Pharmacology, Tufts University School of Medicine, Boston, Massachusetts, United States of America; 2 Sackler School of Graduate Biomedical Sciences, Tufts University, Boston, Massachusetts, United States of America; 3 Department of Biochemistry, Tufts University School of Medicine, Boston, Massachusetts, United States of America; 4 Department of Pharmacy, Tufts Medical Center, Boston, Massachusetts, United States of America; 5 Thorne Research, Inc., Sandpoint, Idaho, United States of America; 6 Vitamin K Lab, Jean Mayer USDA Human Nutrition Research Center on Aging, Tufts University, Boston, Massachusetts, United States of America; 7 First Department of Dermatology, A. Sygros Hospital, Athens University Medical School, Athens, Greece; 8 Department of Internal Medicine, Tufts University School of Medicine and Tufts Medical Center, Boston, Massachusetts, United States of America; Leiden University Medical Center, The Netherlands

## Abstract

Mast cells are immune cells critical in the pathogenesis of allergic, but also inflammatory and autoimmune diseases through release of many pro-inflammatory cytokines such as IL-8 and TNF. Contact dermatitis and photosensitivity are skin conditions that involve non-immune triggers such as substance P (SP), and do not respond to conventional treatment. Inhibition of mast cell cytokine release could be effective therapy for such diseases. Unfortunately, disodium cromoglycate (cromolyn), the only compound marketed as a mast cell “stabilizer”, is not particularly effective in blocking human mast cells. Instead, flavonoids are potent anti-oxidant and anti-inflammatory compounds with mast cell inhibitory actions. Here, we first compared the flavonoid quercetin (Que) and cromolyn on cultured human mast cells. Que and cromolyn (100 µM) can effectively inhibit secretion of histamine and PGD_2_. Que and cromolyn also inhibit histamine, leukotrienes and PGD_2_ from primary human cord blood-derived cultured mast cells (hCBMCs) stimulated by IgE/Anti-IgE. However, Que is more effective than cromolyn in inhibiting IL-8 and TNF release from LAD2 mast cells stimulated by SP. Moreover, Que reduces IL-6 release from hCBMCs in a dose-dependent manner. Que inhibits cytosolic calcium level increase and NF-kappa B activation. Interestingly, Que is effective prophylactically, while cromolyn must be added together with the trigger or it rapidly loses its effect. In two pilot, open-label, clinical trials, Que significantly decreased contact dermatitis and photosensitivity, skin conditions that do not respond to conventional treatment. In summary, Que is a promising candidate as an effective mast cell inhibitor for allergic and inflammatory diseases, especially in formulations that permit more sufficient oral absorption.

## Introduction

Mast cells derive from hematopoietic progenitors and mature in tissues depending on microenvironmental conditions [1], [2]. Mast cells are important effector cells in allergic reactions [3]–[6] by secreting histamine, leukotrienes (LTs), prostaglandin D2 (PGD_2_), proteolytic enzymes and several multifunctional cytokines, such as interleukin-6 (IL-6), IL-8, IL-13, tumor necrosis factor (TNF), and vascular endothelial growth factor (VEGF) [7]–[9]. These mediators contribute to the late-phase reactions and to inflammation through the recruitment and activation of immune cells [10], [11]. In addition to IgE and antigen, anaphylatoxins, cytokines, hormones and neuropeptides, such as substance P (SP), can trigger mast cell secretion [12] of several mediators often selectively [13]. More importantly, SP has been implicated in many skin inflammatory disorders, including contact dermatitis [14], [15]. As a result, mast cells are also involved in innate and acquired immunity [16], as well as autoimmunity and inflammation [12], especially in the skin [17], [18]. Mast cells were also shown to be involved in contact dermatitis in mice [19], [20].

Mast cells play an essential role in contact hypersensitivity through a complex interaction with different kinds of immune cells, including antigen presenting cells, T, B, NK lymphocytes, keratinocytes, endothelium, and platelets [21]. In allergic contact dermatitis, mast cells regulate the inflammatory reactions by releasing mediators like histamine, TNF-α and IL-8, leading to local vascular activation and subsequent immune cell recruitment [22]. Contact dermatitis and photosensitivity are difficult to treat with conventional treatments [23], [24], and disodium cromoglycate (cromolyn) has failed in these diseases [25]. Several studies even reported development of contact dermatitis from cromolyn-containing eyedrops 26,27. Here we compared the ability of quercetin and cromolyn to inhibit key mediators from human cultured mast cells stimulated by SP or IgE/Anti-IgE, as well as the effect of quercetin on contact dermatitis and photosensitivity in humans.

There are no *effective* clinically available mast cell inhibitors. *In vitro*, cromolyn inhibits *rodent* peritoneal mast cell histamine secretion [28], but could not inhibit rat mucosal mast cells even at 1000 µM [29], [30]. Moreover, 1000 µM cromolyn was required for any inhibition of histamine release from human lung and tonsillar mast cells [31]. Even though, cromolyn was ineffective in inhibiting human lung and intestinal mast cells [32]. Cromolyn is used in the management of mastocytosis to relieve diarrhea and abdominal cramping [33]. Intestinal absorption of cromolyn is severely limited (≤1%), and several weeks of therapy may be needed before any clinical benefits are seen [34]. This finding goes against the well known phenomenon of tachyphylaxis exhibited during cromolyn pretreatment [28].

Certain naturally occurring flavonoids, such as quercetin (Fig. S1B), are polyphenolic compounds found in fruits, vegetables, nuts, seeds, herbs, spices and red wine with antioxidant properties [35]–[37]. Flavonoids have potent anti-oxidant, anti-inflammatory [38], and mast cell blocking activities [39], [40]. Several flavonoids can inhibit histamine release from murine mast cells [41], [42], as well as IL-6 and TNF release from bone marrow-derived cultured murine mast cells and rat peritoneal mast cells [43]. Quercetin and its structurally related luteolin inhibit the release of histamine, leukotrienes and PGD_2_ from human cultured mast cells in response to cross-linkage of high affinity surface IgE receptors (FcεRI) [44]. Quercetin also inhibits histamine, IL-6, IL-8, TNF-α and tryptase release from human mast cells [39], [40], as well as asthma development in an animal model [45]. Quercetin could inhibit stimulated rat peritoneal mast cells by 75% at 10 µM, while 1000 µM of cromolyn was required for 65% inhibition [46]. However, quercetin was never compared to cromolyn on human mast cell mediator release in response to either an allergic or non-immune trigger.

Here we show that quercetin is more effective than cromolyn in inhibiting inflammatory cytokine release from human cultured mast cells.

## Results

### Effect on primary hCBMC cell histamine, PGD_2_ and leukotriene release

**Figure 1:**
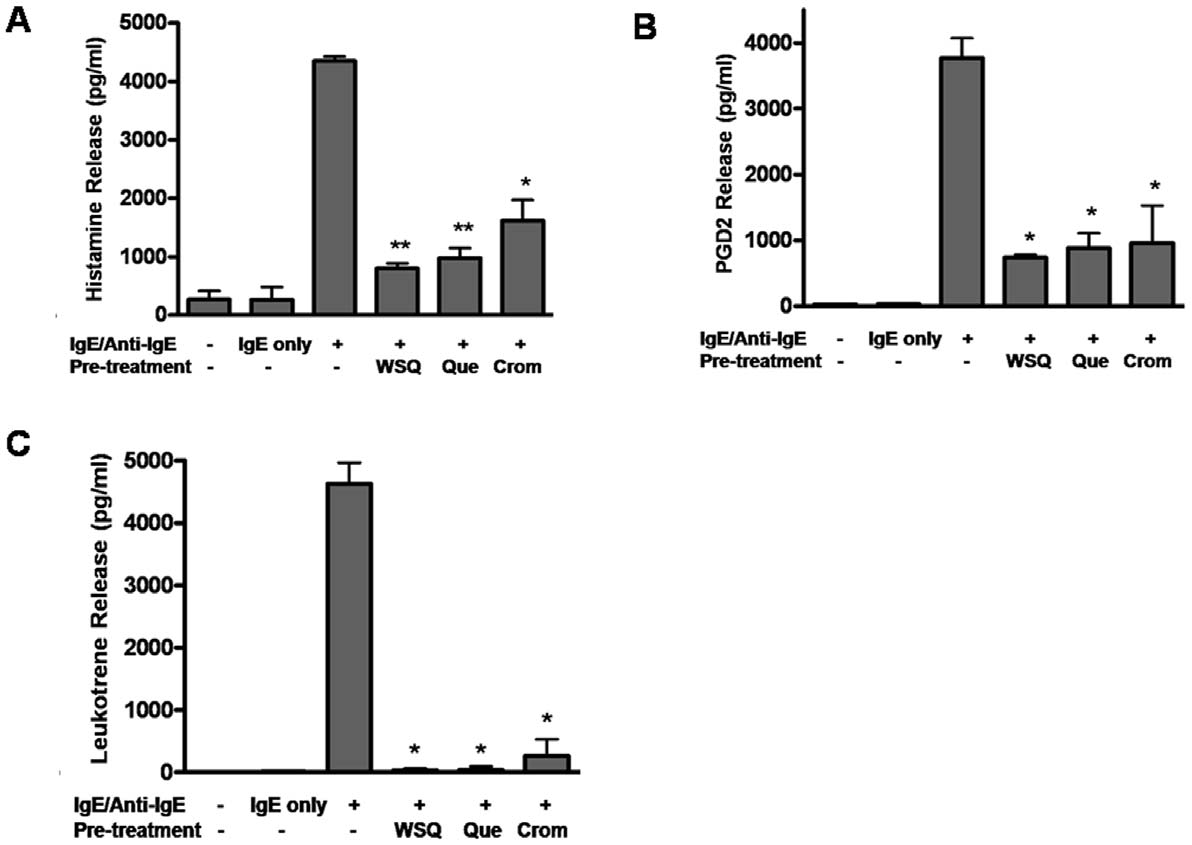
Quercetin and cromolyn inhibit degranulation of hCBMCs triggered by IgE/Anti-IgE. Cells were treated with IgE (100 ng/mL) for 2 hr and then stimulated with Anti-IgE (1 µg/mL) for 2 hr. In some experiments, cells were pre-incubated with Que or WSQ (100 µM, 30 min), or treated with cromolyn (100 µM) together with Anti-IgE. (A) Histamine; (B) PGD_2_; (C) Leukotriene. n = 3, * indicates p<0.05, ** indicates p<0.01. WSQ = water soluble quercetin; Que = quercetin; Crom = cromolyn.

Stimulation of hCBMCs with IgE/Anti-IgE results in rapid secretion of large amounts of histamine, PDG2 and LTs (Fig. 1A–C). Preincubation with Que or WSQ (100 µM) significantly reduce histamine secretion (Fig. 1A) from 4347.9 to 977.3 pg/mL (82% inhibition), and to 797.7 pg/mL (87% inhibition), respectively, compared to cromolyn (1613.6 pg/mL, 67% inhibition). Que and WSQ inhibit PGD_2_ release (Fig. 1B) from 3771.8 to 882.5 pg/mL (77% inhibition), and to 740.8 pg/mL (81% inhibition), respectively, compared to cromolyn (958.3 pg/mL, 75% inhibition). In addition, Que and WSQ block LT secretion (Fig. 1C) from 4628.6 to 35.3 pg/mL (99% inhibition), and to 29.7 pg/mL (99% inhibition), respectively, compared to cromolyn (530.9 pg/mL, 88% inhibition). Cells were >95% viable after 24 hr incubation with the drugs (Fig. S2A). Que was effective when added 30 min prior to the trigger. No significant inhibitory effect was observed when Que was added together with the trigger (Fig. S3A–C). However, cromolyn had to be added **together** with the trigger for any inhibitory effect to be evident (Fig. S4).

### Effect on human mast cell histamine, IL-8, TNF and IL-6 release

**Figure 2:**
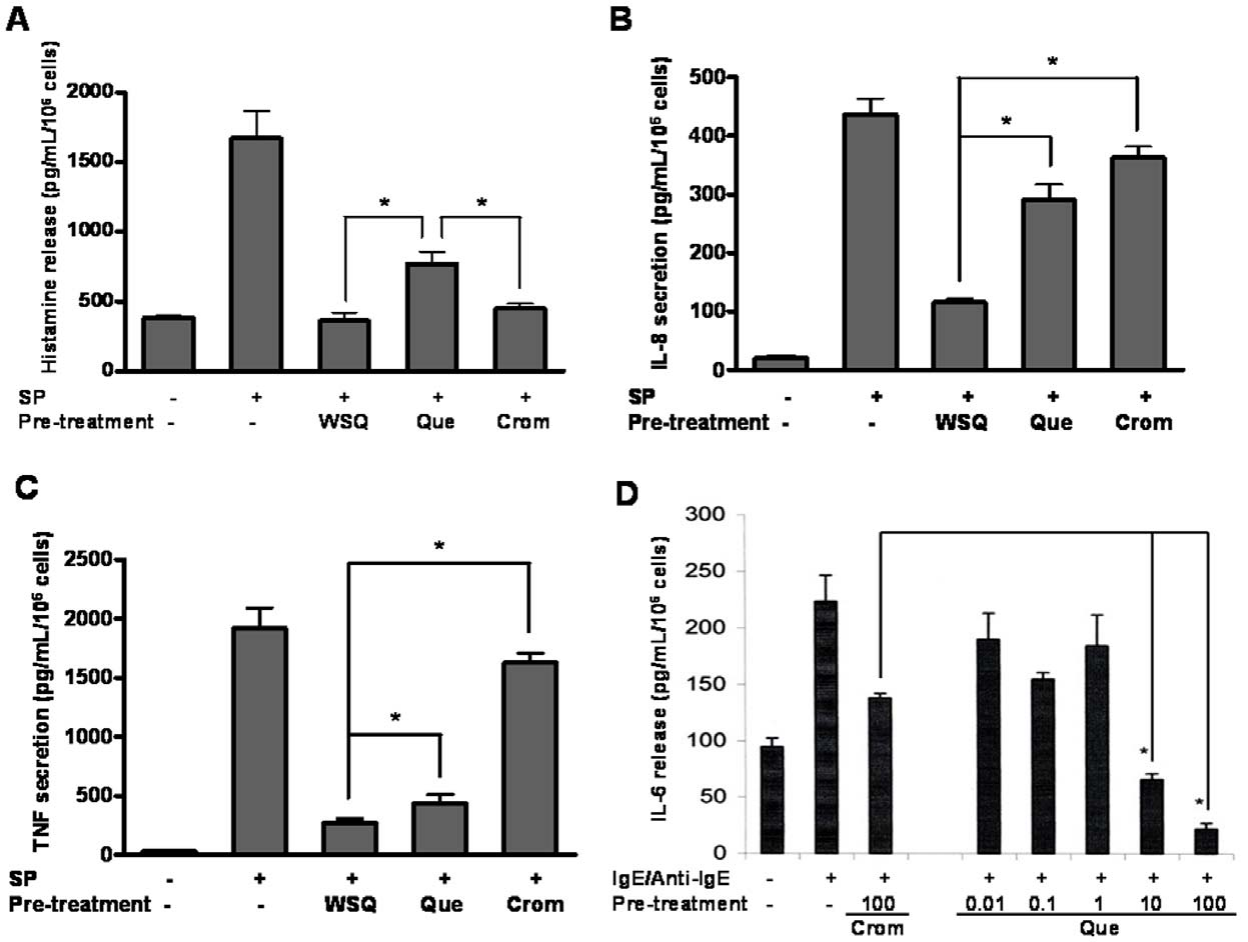
Quercetin inhibits SP-triggered mediator release from human mast cells. LAD2 cells were stimulated with SP (2 µM). In some experiments, cells were pre-incubated with Que or WSQ (100 µM, 30 min), or treated with cromolyn (100 µM) together with SP. (A) Histamine; (B) IL-8; (C) TNF. In (D) IL-6; hCBMCs were treated with either 100 µM cromolyn together with the trigger, or pre-incubated with increasing concentrations of quercetin for 30 min, then stimulated with IgE/Anti-IgE as described in Fig. 1. Histamin release was measured at 2 hr. Cytokine release was measured at 24 hr. n = 3, * indicates p<0.05.

LAD2 mast cells release histamine 2 hr after SP (2 µM) stimulation (1611.4 pg/mL, Fig. 2A). WSQ inhibits histamine release to 350.6 pg/mL, which is even lower than that of control (388.7 pg/mL). Quercetin and cromolyn reduce histamine release to 792.3 pg/mL and 464.6 pg/mL, respectively (Fig. 2A). LAD2 mast cells also secrete newly-synthesized IL-8 and TNF 24 hr after SP (2 µM) stimulation. Incubation with WSQ, quercetin and cromolyn (100 µM) effectively block secretion of IL-8 from 437.2 pg/mL to 115.4 pg/mL, 291.2 pg/mL and 362.9 pg/mL, respectively. Similarly, WSQ, quercetin and cromolyn (100 µM) block TNF secretion from 1917.2 pg/mL to 274.7 pg/mL, 436.9 pg/mL and 1628.8 pg/mL, respectively (Fig. 2B&C). WSQ is the most effective while cromolyn is the least effective.

Primary hCBMCs release IL-6 (223.5 pg/mL) 24 hr after stimulation with IgE/Anti-IgE, and is inhibited by quercetin in a dose-dependent manner (Fig. 2D). Quercetin preincubation at 10 and 100 µM for 30 min reduces IL-6 release to 65.8 pg/mL and 29.7 pg/mL, respectively, which is even lower than that of control (96.8 pg/mL, Fig. 2D). Cromolyn (100 µM) is able to decrease IL-6 release to 141.3 pg/mL.

It should be noted again that, when added together with the trigger, quercetin does not exhibit any significant inhibitory effect on SP-triggered mediator release from LAD2 cells (Fig. S3D–F). After 30 min preincubation, cromolyn has no effect due to rapid tachyphylaxis (Fig. S4). Instead, cromolyn had to be added **together** with SP for any inhibitory effect to be evident. Cells were >95% viable after 24 hr incubation with the drugs (Fig. S2B).

### Effect on intracellular calcium increase in human mast cells

**Figure 3:**
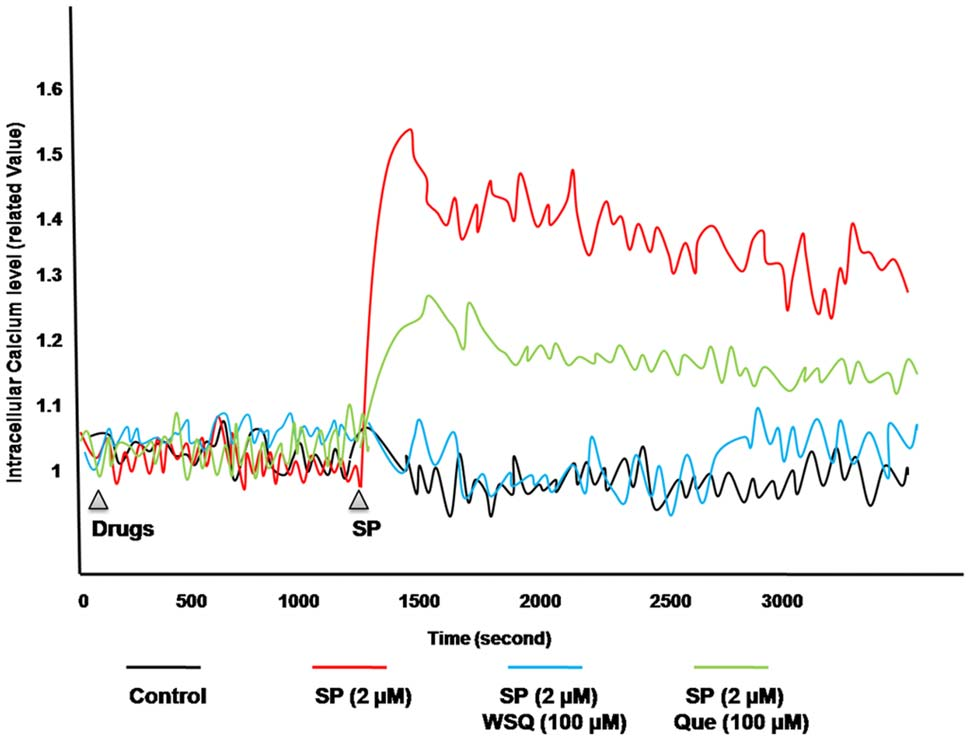
Quercetin inhibits intracellular calcium levels in SP-stimulated LAD mast cells. Cells were loaded with Fura-2 AM (30 nM, 20 min) and then pre-incubated with flavonoids (100 µM, 30 min). Immediately after SP addition (2 µM), Fura-2 AM fluorescence was detected for 20 min.

In order to investigate the possible mechanism of action of these agents, we studied the effect on intracellular calcium ions. Preincubation with WSQ (100 µM, 30 min) completely inhibits the intracellular calcium ion increase as compared to equimolar concentration of quercetin (Fig. 3), which is half as active. Cromolyn (100 µM) has no effect on calcium levels even added together with the trigger (data not shown).

### Effect on NF-κB nuclear translocation in LAD2 mast cells

**Figure 4:**
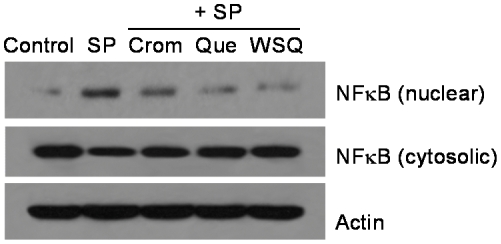
Quercetin inhibits nuclear translocation of NF-κB in LAD2 mast cells. Cells were pretreated with drugs (100 µM, 30 min) and then stimulated by SP (2 µM, 60 min). Upper lane, NF-κB protein levels in nuclear fractions before and after SP stimulation; Middle lane, NF-κB protein levels in cytosolic fractions before and after SP stimulation.

Cytocolic NF-κB protein levels do not change much before and after SP stimulation (Fig. 4). SP induces NF-κB translocation into the nucleus within 60 min compared to control. This NF-κB nuclear translocation is inhibited by 30 min preincubation with WSQ, which is more effective than quercetin and cromolyn (Fig. 4).

### Effect on contact dermatitis and skin photosensitivity in humans

**Figure 5:**
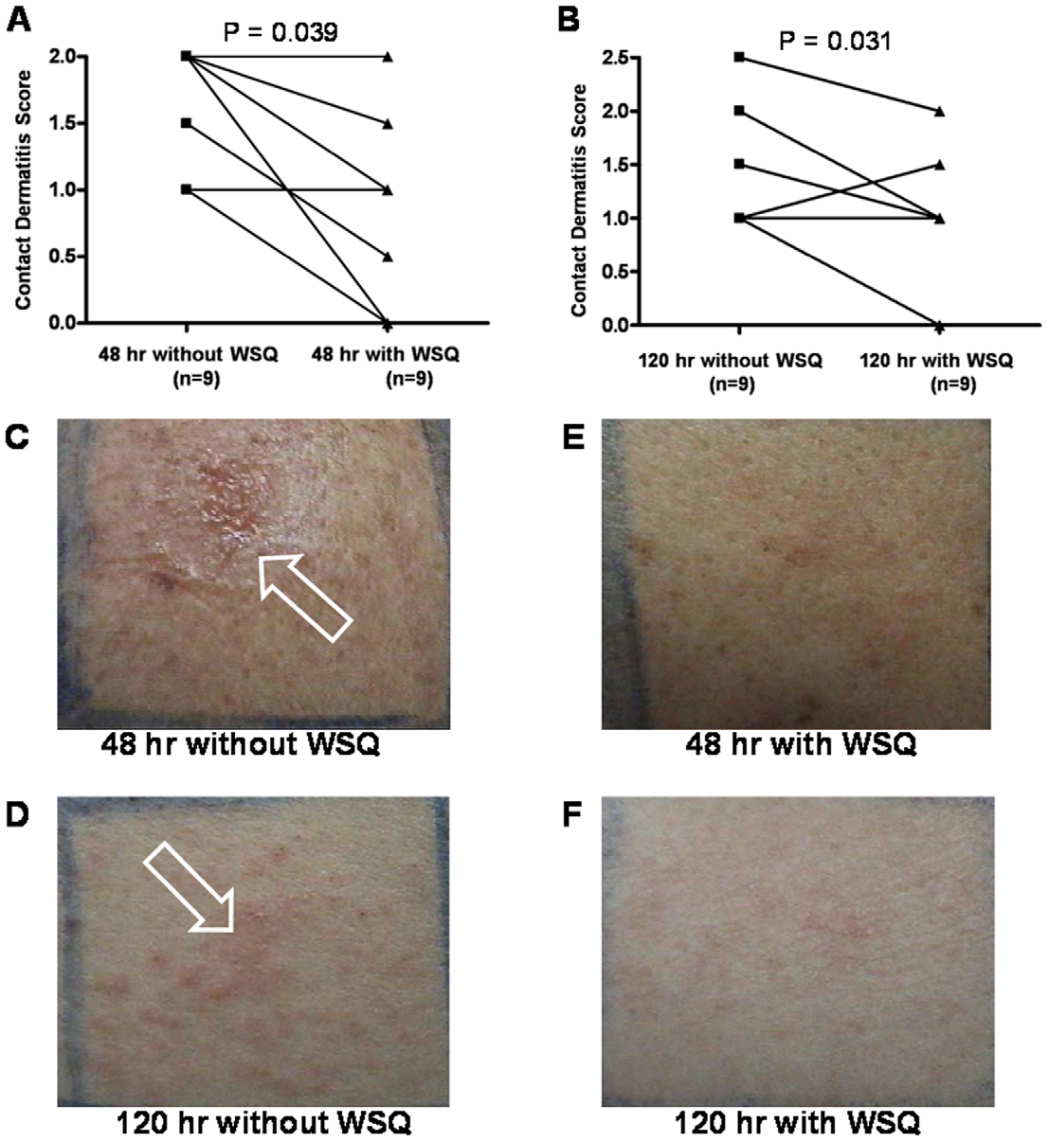
Inhibitory effect of WSQ on nickel patch tests. Examination of patch tests was done at 48 hr and 120 hr after application of the patch containing nickel first without WSQ and then one week later, 3 days after having taken WSQ (2 g/day). (A&B) Individual patient data from nickel patch tests were analyzed by the Wilcoxon paired non-parametric test. Representative patch test from patient No. 8 is shown in C–F. Appearance at (C) 48 hr or (D) 120 hr without WSQ. (E) 48 hr or (F) 120 hr during which patient was administered WSQ. White arrows indicate the area of nickel patch test. It should be noted that associated pruritus (itching) was eliminated in all subjects, along with widespread itching in one patient who also had chronic atopic dermatitis.

Patch tests conducted with nickel in 10 volunteers showed extensive dermatitis ranging from 1+ to 3+ at 48 hr and 120 hr after nickel contact (Fig. 5A&B). Pre-administraiton of WSQ (2 g/day for 3 days) before nickel contact on the same volunteers effectively reduced this reaction by more than 50% in 8 out of 10 patietns, and 100% in the other 2 patients (Fig. 5A&B, Wilxocon paired non-parametric test, p = 0.039 for 48 hr, and p = 0.031 for 120 hr after nickel contact). Moreover, any associated pruritus disappeared as did the generalized pruritus present in one patient with pre-exsiting atopic dermatitis.

**Figure 6:**
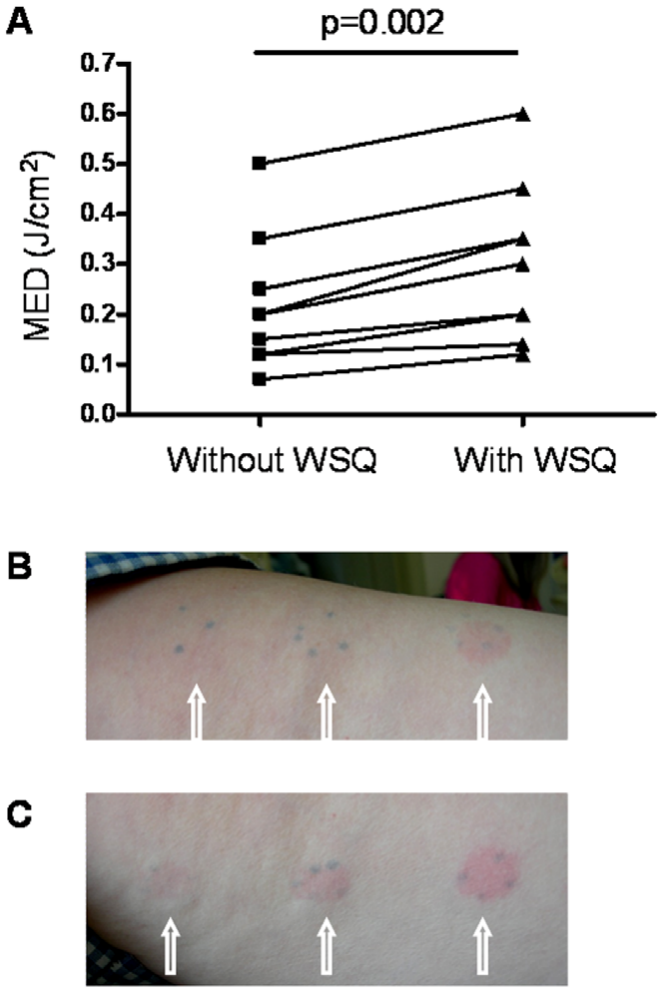
Inhibitory effect of WSQ on skin photosensitivity. (A) Energy fluence values at MED estimated 24 hr after irradiation with and without WSQ (1 g). Data was analyzed by Wilcoxon paired non-parametric test. Photos show three difference spots on the medial aspect of the forearm, representing three different energy fluence values of UVB broad band irradiation (UVB 801, Waldmann, 3.03 mW at 21 cm). (B) Skin appearance 24 hr after irradiation with WSQ administration (1 g). (C) Skin appearance 24 hr after irradiation without WSQ administration. White arrows indicate spots of UVB irradiation.

As shown in Fig. 6, administration of 1 g WSQ increases the MED to induce skin erythema 24 hours after irradiation in all patients (Wilcoxon paired non-parametric test, p = 0.002). MED is defined as the minimal dose that induces any visible reddening. An increase in MED demonstrates increased resistance to develop skin reddening after UVB irradiation.

## Discussion

Here we report on the comparative inhibitory effect of WSQ, Que and the “mast cell stabilizer” cromolyn on human mast cell secretion of inflammatory mediators. Que is equally effective to cromolyn in inhibiting histamine, PGD_2_ and LT release but much more effective in reducing cytokine release from human mast cells.

The limited number of mast cells obtained from normal human tissues has led to increasing use of human leukemic cultured LAD2 cells [47] or human umbilical cord blood-derived cultured mast cells (hCBMCs) previously reported to release cytokines, histamine and tryptase in response to anti-IgE [48], [49]. In the present study, we used IgE/Anti-IgE as a positive mast cell trigger for hCBMCs, and SP for LAD2 cells. We chose to do so because recent studies have implicated sensory neuropeptides, such as SP, in the pathogenesis of many skin diseases, including contact dermatitis [14], [15].

Cromolyn had previously been reported to inhibit histamine secretion from rodent peritoneal mast cells, but was a much weaker inhibitor of human mast cells [31], [50]. Moreover, cromolyn does not inhibit lung and mucosal mast cells [31], [32]. Cromolyn was previsouly shown to inhibit TNF release from rat peritoneal mast cells by only 20% at 100 µM [51] and 1000 µM concentration was reported for any inhibition of human lung mast cells [31]. Clinically, cromolyn has been used in the management of mastocytosis [33], [52] and other allergic diseases, including atopic dermatitis [53], [54]. Although mast cell stabilization was initially considered as its mechanism of action, modulation of sensory nerve fiber activation has also been suggested. For instance, topical cromolyn treatment effectively reduces allergen-induced pruritus but has no effect on weals, supporting that in the skin, cromolyn inhibits sensory C-fiber nerve activation rather than inhibiting mast cell degranulation [55]. Instead, Que and luteolin were shown to inhibit histamine, IL-6 and TNF production from bone-marrow derived cultured murine mast cells [43]. Quercetin had also been reported to inhibit histamine release from rat connective tissue mast cells and mucosal mast cells [56], as well as from human lung and intestinal mast cells [32].

Here we also show that WSQ is more effective than quercetin in blocking intracellular calcium increases in stimulated human mast cells, while cromolyn had no effect. WSQ and Que are appear to be more effective than cromolyn in inhibiting NF-κB nuclear translocation, which is necessary for the production of pro-inflammatory cytokines. However, our results are not quantitative because we did not perform a reporter gene assay. Previous studies had reported that activation of hCBMCs by anti-IgE increased intracellular calcium levels [48], [57], a necessary step for mediator release [58]. Fewtrell and Gomperts first showed that Que could inhibit calcium influx in rat peritoneal mast cells [59]. Previous reports showed that IgE-mediated release of histamine and leukotrienes was abolished after calcium depletion [60]. Que also inhibited ionophore-induced histamine release from rat peritoneal mast cells, suggesting it had actions other than receptor-mediated calcium influx [46]. The structure of Que has some similarity to that of cromolyn (Fig. S1), but their actions appear to differ considerably. Not only is Que more effective than cromolyn, but Que inhibits intracellular calcium increases, while cromolyn does not. An additional important difference is that Que is effective prophylactically, but not when added together with the trigger, while cromolyn shows inhibition **only when added together with the trigger**. The lack of cromolyn's inhibitory action when used prophylactically was reported before and was termed tachyphylaxis. In fact, cromolyn's inhibitory action and tachyphylaxis were related to the phosphorylation of moesin [61], [62], which also showed tachyphylaxis with cromolyn pre-incubation in stimulated rat peritoneal mast cells [28], [62]. Que also stimulated phosphorylation of moesin in rat peritoneal mast cells [63].

Here we report for the first time to our knowledge that Que can be beneficial in contact dermatitis and photosensitivity in humans, conditions that are typically difficult to treat [64], [65]. Cromolyn was not tried in either one of these two models. There is only one publication from 1990 where patients with nickel-positive pompholyx (skin reactions even in the absence of contact with nickel) “reacted better” after prolonged (15 days) treatment with oral cromolyn [66]. The *in vitro* concentration of cromolyn we used (100 µM) is approximately 10 times more than the concentration administered in humans assuming even distribution of 400 mg oral cromolyn (molecular weight 460) in a 80 kg human per day. The *in vitro* concentration of quercetin (molecular weight 300) we used (100 µM) is almost identical to that administered to humans (1 g/day) in these two pilot studies.

The apparent beneficial effect of quercetin in these skin conditions could be extended to atopic dermatitis/eczema and psoriasis that are also difficult to treat and involve both neuropeptides and mast cells [67], [68]. The burden of these diseases to the economy is estimated at about $ 5 billion/year. Mast cells have been implicated in allergic and inflammatory diseases of the skin [17], [18]. Contact dermatitis involves both mast cells and T cells [22]. In this context, it is interesting that mast cells were reported to stimulate activated T cells [69], [70], one effect requiring TNF and blocked by the flavone luteolin [69]. Moreover, mast cells have the ability to present antigen [71] and to regulate dendritic cell trafficking through TNF [72]. In fact, mast cell is the only cell type that stores pre-formed TNF [12].

Skin photosensitivity is a rapid reaction that is due to both keratinocyte, mast cell and sensory nerve activation [73], [74]. In this context, it is important to mention that mast cells are in close contact with sensory nerve endings in the skin [75], and can be activated by sensory neuropeptides [76]. In turn, mast cell-derived mediators such as histamine and TNF can stimulate sensory nerve endings [77], [78].

Flavonoids had been reported to improve allergic symptoms or prevent the development of allergic diseases [79], [80]. Moreover, oral administration of astragalin, which is absorbed and converted into kaempferol, suppresed the onset of dermatitis in NC/Nga mice [81]. In a phase I clinical trial of Que, a dose of 2.5 g for a 70 kg individual administered via intravenous infusion at 3-week or weekly intervals was recommended for Phase II trials [82]. The Que dose used in humans in the present study (2 g/day) is clinically relevant. A major drawback of the use of Que and cromolyn in the clinical setting is their poor water solubility. Previsous studies showed that the oral absorption of quercetin and cromolyn is only 1% [83] and 1–5% [84] respectively. WSQ is soluble in aqueous medium only, but is likely to precipitate in the acidic environment of the stomach while used *in vivo*. A liposomal or enteric coated formulation may offer advantage for increased bioavailability. A phytoquercetin is now under development and appears to have much higher oral absorption.

In conclusion, our results indicate that quercetin is more effective than cromolyn in inhibiting release of pro-inflammatory cytokines from human mast cells. Moreover, quercetin appears to significantly reduce contact dermatitis, photosensitivity and associated pruritus that are typically resistant to anti-histamines and cromolyn [85], [86]. Quercetin is safe [87] and could be particularly useful in formulations that increase its solubility and oral absorption.

## Materials and Methods

### Drugs and Reagents

Recombinant human stem cell factor (rhSCF) was kindly donated by Biobitrum AB, (Stockholm, Sweden). Cromolyn, quercetin and SP were obtained from Sigma-Aldrich (St Louis, MO). A sodium salt of quercetin soluble in water at pH 7.4 or higher (WSQ) was provided by Thorne Research, Inc. (Dover, ID). All drugs were dissolved in DMSO, except for WSQ, which was dissolved in double-distilled water forming a solution of pH≈9. Final concentration of DMSO was <0.1% and pH was 7.4. Rabbit anti-NF-κB (p65, RelA) (Ab-1) antibody was purchased from Millipore (Billerica, MA), rabbit anti-actin antibody and the secondary HRP-conjugated antibody were purchased from Cell Signaling Technology (Beverly, MA).

### Human mast cell culture

LAD2 human mast cells (obtained from Dr. A Krisherbaum, NIH) were cultured in serum-free media (StemPro-34, Gibco, Grand Island, NY) supplemented with 2 mM L-glutamine, 100 IU/mL penicillin, 50 µg/mL streptomycin, and 100 ng/mL rhSCF. For optimal cell growth, LAD2 cell density was maintained between 0.5×10^6^ and 1×10^6^ cells/mL.

In order to obtain primary human umbilical cord blood-derived cultured mast cells (hCBMCs), human cord blood was obtained from placenta during normal deliveries at Tufts Medical Center in accordance with established institutional guidelines [88]. Briefly, mononuclear cells were isolated by layering heparin-treated cord blood onto Lymphocyte Separation Medium (INC Biomedical, Aurora, OH). CD34+ progenitor cells were isolated from mononuclear cells by positive selection of AC133 (CD133+/CD34+) cells by magnetic cell sorting (Miltenyi Biotech, Auburn, CA). hCBMCs were derived by the culture of CD34+ progenitor cells with minor modifications. For the first six weeks, CD34+ cells were cultured in AIM medium (Gibco, Grand Island, NY) supplemented with 100 ng/mL rhSCF, after six weeks of culture, 50 ng/mL IL-6 (Chemicon) was added. Cells were cultured at 37°C in 5% CO_2_ balanced air. Mast cell viability was determined by trypan blue (0.3%) exclusion and was better than 98%.

### Mast cell stimulation

LAD2 cells were washed with DPBS and resuspended in complete culture medium. LAD2 cells (1×10^5^ cells/200 µL/well) were plated in 96 well flat bottom Falcon cell culture plates from Becton Dickinson (Franklin Lakes, NJ), and then stimulated with SP (2 µM) for 24 hr at 37°C in 5% CO_2_ incubator. Primary hCBMCs (1×10^5^ cells/200 µL/well) were stimulated with IgE (100 ng/mL) for 2 hr followed by Anti-IgE (1 µg/mL) for another 2 hr. Control cells were treated with equal volume of culture medium only. The supernatant fluid was collected for further assays.

### Degranulation assays

Mast cell degranulation was assessed by measuring histamine, PGD_2_ and LTs release in the supernatant fluid 30 min after cell stimulation. Histamine levels were assayed using a SPI Bio histamine EIA kit (Bertin Pharma, France). PGD_2_ release was measured using a PGD_2_-MOX EIA kit (Cayman Chemical Co., Ann Arbor, MI). LTs levels were assayed using a Luminex cysteinyl leukotriene kit (Cayman Chemical Co.) according to the manufacturer's instructions.

### TNF and IL-8 release assays

IL-8 and TNF release into the supernatant fluid 24 hrs after cell stimulation were measured by Enzyme-Linked Immunosorbent Assay (ELISA) using a commercial kit from R&D Systems (Minneapolis, MN) as per the instructions. The minimum detectable level of IL-8 and TNF was 5 pg/mL.

### Cytosolic calcium measurements

Cytosolic calcium was measured at 37°C using Fura-2 as indicator. LAD2 cells were loaded in Tyrode's buffer with 30 nM Fura-2 AM (Invitrogen) for 20 min to allow Fura-2 to enter the cells. After centrifugation to remove excess dye, the cells were resuspended in Tyrode's buffer at a concentration of 10^6^ cells/mL and incubated for another 20 min. Cells were then transferred to 96-well plates with 100 µL per well. SP (2 µM) was added to cells for the time indicated. Fura-2 fluorescence was read by MDC FlexStation II (Molecular Devices) at an excitation wavelength of 340 nm/380 nm and emission wavelength of 510 nm. Results were processed according to the Invitrogen Fura-2 protocol and reported as relative value of OD 340/380.

### NF-κB nuclear translocation assay

After preincubation with different drugs (100 µM, 30 min), LAD2 mast cells (2×10^5^ cells per well) were stimulated with SP (2 µM) for 60 min. Nuclear fractions were isolated using a NE-PER nuclear extraction kit (Thermo Scientific, Rockford, IL). Changes in NF-κB protein levels in both cytosolic and nuclear fractions were detected by Western blot analysis. The protein concentrations were determined using Bio-Rad Protein Assay reagent, and equal amounts of protein were subjected to Western blotting by using the indicated antibodies. Briefly, samples separated by SDS/PAGE were transferred to nitrocellulose membranes. After being blocked in 5% BSA (w/v) at room temperature for 1 hr, the membranes were rinsed and incubated at 4°C overnight with a variety of primary antibodies (1∶1,000 dilution). The membranes were then washed and incubated with secondary antibody (1∶2,000 dilution) at room temperature for 1 hr, developed with chemiluminescence ECL reagent (LumiGold, SignaGen Laboratories, Gaithersburg, MD), and exposed to Hyperfilm MP (GE Healthcare, Piscataway, NJ).

### Nickel-contact dermatitis in human subjects

Patch tests were performed to volunteers (n = 10, all female, age 30–42 years old) sensitive to contact with nickel before and after taking 2 g/day WSQ (4×500 mg/day) orally for 3 days. The patch containing nickel was first applied without pre-treatment of WSQ. Examination of the skin site of patch tests was done and photographs obtained at 48 and 120 hr after application of the patch. After one-week wash-out period, the same subjects were given 3 days WSQ as noted above before applying the patch again to the same arm a few cm distance from the previous site. Examination of the skin site of patch tests was again done at 48 and 120 hr after application of the patch. Results were recorded as scores ranging from 1 to 3, with 1 being the least affected and 3 being the worst affected condition. Skin sites were analyzed with no identifiers. Written consents have been obtained from all participants. This protocol was approved by the Human Ethics Review Committee of A. Sygros Hospital of Athens University Medical School.

### Skin photosensitivity in human subjects

Minimal erythema dose (MED) that induces visible reddening was measured in the medial aspect of the forearm in three different spots (∼2 cm^2^) of different power fluence values of UVB broad band irradiation (UVB 801, Waldmann, 3.03 mW at 21 cm). First, the skin was irradiated without WSQ administration. Skin erythema was evaluated and photographed at 24 hr after irradiation. After one-week wash-out period, the same subjects were irradiated at three skin spots close to the previous irradiated sites with MED and higher power fluence values of UVB broad band irradiation 2 hr after administration of 1 g WSQ (4×250 mg) orally. Skin erythema was again evaluated and photographed 24 hr after irradiation. All subjects were females, age 42.6±12.2 years. Skin erythema was evaluated with no identifiers. Written consents have been obtained from all participants. This protocol was approved by the Human Ethics Review Committee of A. Sygros Hospital of Athens University Medical School.

### Statistical analysis

All *in vitro* conditions were performed in triplicates, and all experiments were repeated at least three times (n = 3). Results are presented as mean±SD. The results on mediator release are presented as percent inhibition to normalize baseline differences between cultures that might be due to variation among the individual batches of hCBMCs obtained from different donors, as previously reported [89], [90]. Data from stimulated and control samples were compared using the unpaired, two-tailed, Student's *t*-test. The *in vivo* patient data were analyzed using two-tailed, Wilxocon matched pairs test with representative photos showing affected and unaffected sites. Significance of comparisons is denoted by p<0.05.

## Supporting Information

Figure S1 **Structures of cromolyn and quercetin.** Circles indicate the structural similarity between cromolyn and quercetin. Rectangle indicates the hydroxyl group critical for inhibitory activity.
(TIF)

Figure S2 **Viability of hCBMC and LAD2 mast cells.** hCBMC (A) and LAD2 (B) cells were treated with different drugs as indicated in Fig. 1 & 2 for 24 hr. Cell viability was checked using the Trypan Blue exclusion test. Numbr of viable cells are presented as a percentage of the total cell number. n = 3.
(TIF)

Figure S3 **Quercetin loses its inhibitory effects on human mast cells when added together with the trigger.** Human mast cells were stimulated as described in Fig. 1 & 2. In some experiments, cells were treated with WSQ or Que (100 µM) together with the trigger without pre-incubation. (A–C) hCBMCs; (D–F) LAD2. n = 3.
(TIF)

Figure S4 **Cromolyn does not show significant inhibition on human mast cells when added 30 min prior to the trigger.** Human mast cells were stimulated as described in Fig. 1 & 2. In some experiments, cells were treated with Crom (100 µM) 30 min prior to the trigger. (A–C) hCBMCs; (D–F) LAD2. n = 3.
(TIF)
