# Supplementary figures and images for: Quercetin Is More Effective than Cromolyn in Blocking Human Mast Cell Cytokine Release and Inhibits Contact Dermatitis and Photosensitivity in Humans
Source: PLoS One. 2012 Mar 28;7(3):e33805. doi: 10.1371/journal.pone.0033805 (PMC3314669; doi:10.1371/journal.pone.0033805)

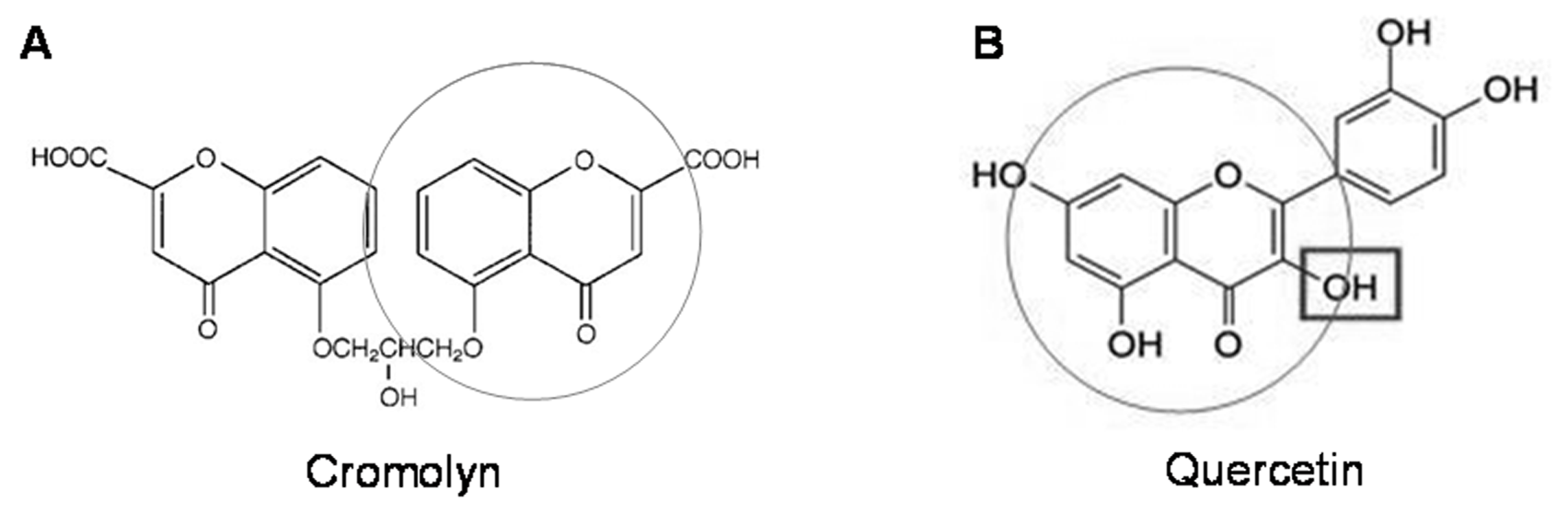

Supplement: Figure S1 — Structures of cromolyn and quercetin. Circles indicate the structural similarity between cromolyn and quercetin. Rectangle indicates the hydroxyl group critical for inhibitory activity. (TIF) [file pone.0033805.s001.tif]

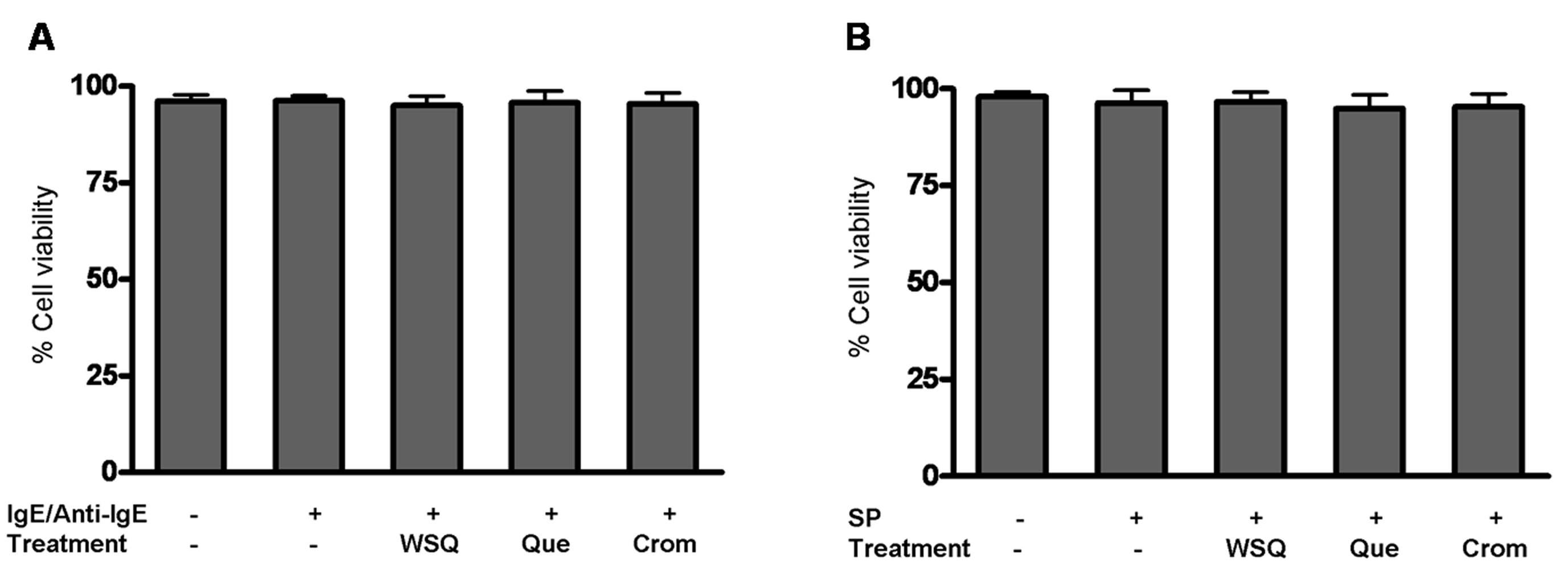

Supplement: Figure S2 — Viability of hCBMC and LAD2 mast cells. hCBMC (A) and LAD2 (B) cells were treated with different drugs as indicated in Fig. 1 & 2 for 24 hr. Cell viability was checked using the Trypan Blue exclusion test. Numbr of viable cells are presented as a percentage of the total cell number. n = 3. (TIF) [file pone.0033805.s002.tif]

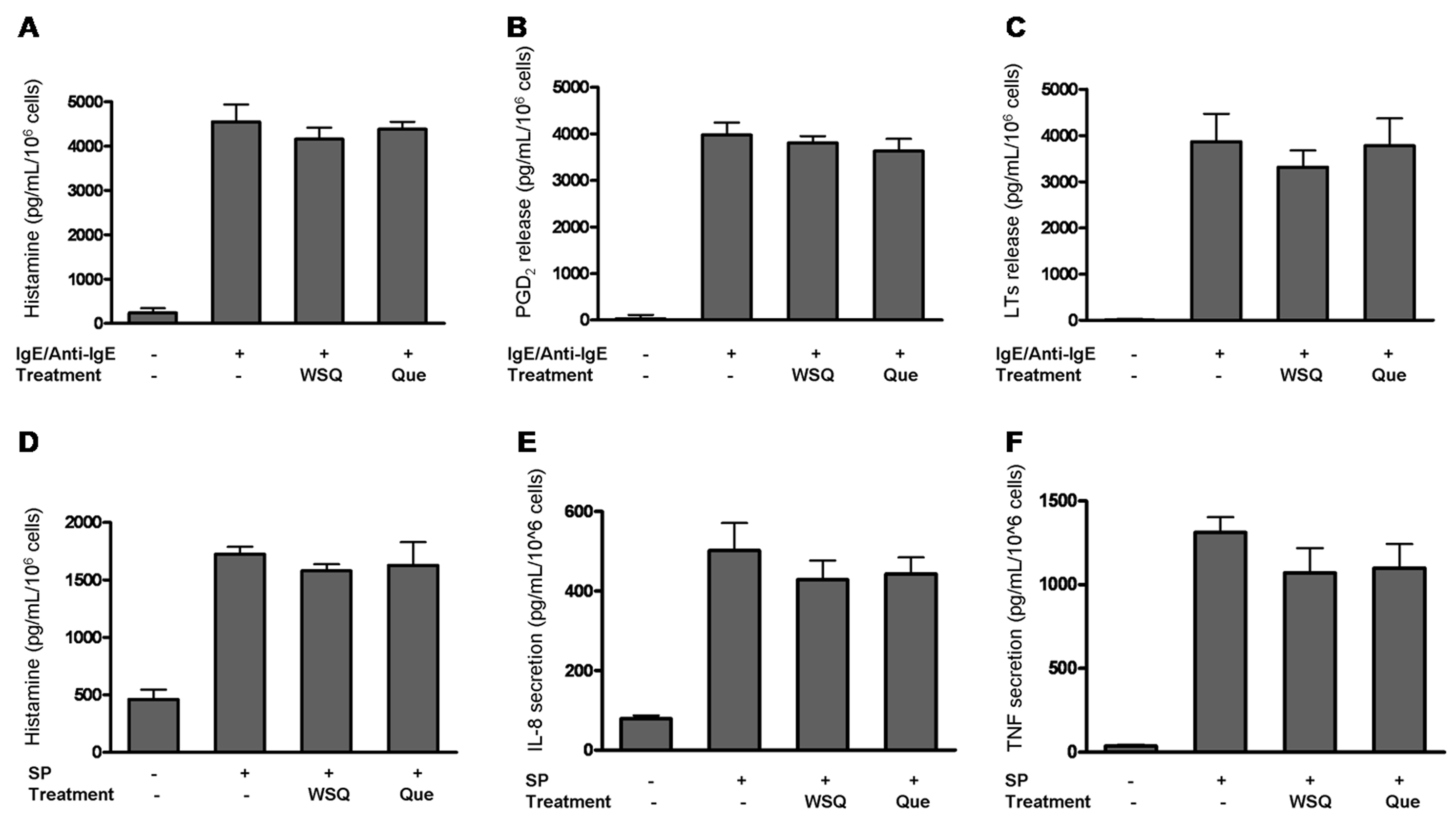

Supplement: Figure S3 — Quercetin loses its inhibitory effects on human mast cells when added together with the trigger. Human mast cells were stimulated as described in Fig. 1 & 2. In some experiments, cells were treated with WSQ or Que (100 µM) together with the trigger without pre-incubation. (A–C) hCBMCs; (D–F) LAD2. n = 3. (TIF) [file pone.0033805.s003.tif]

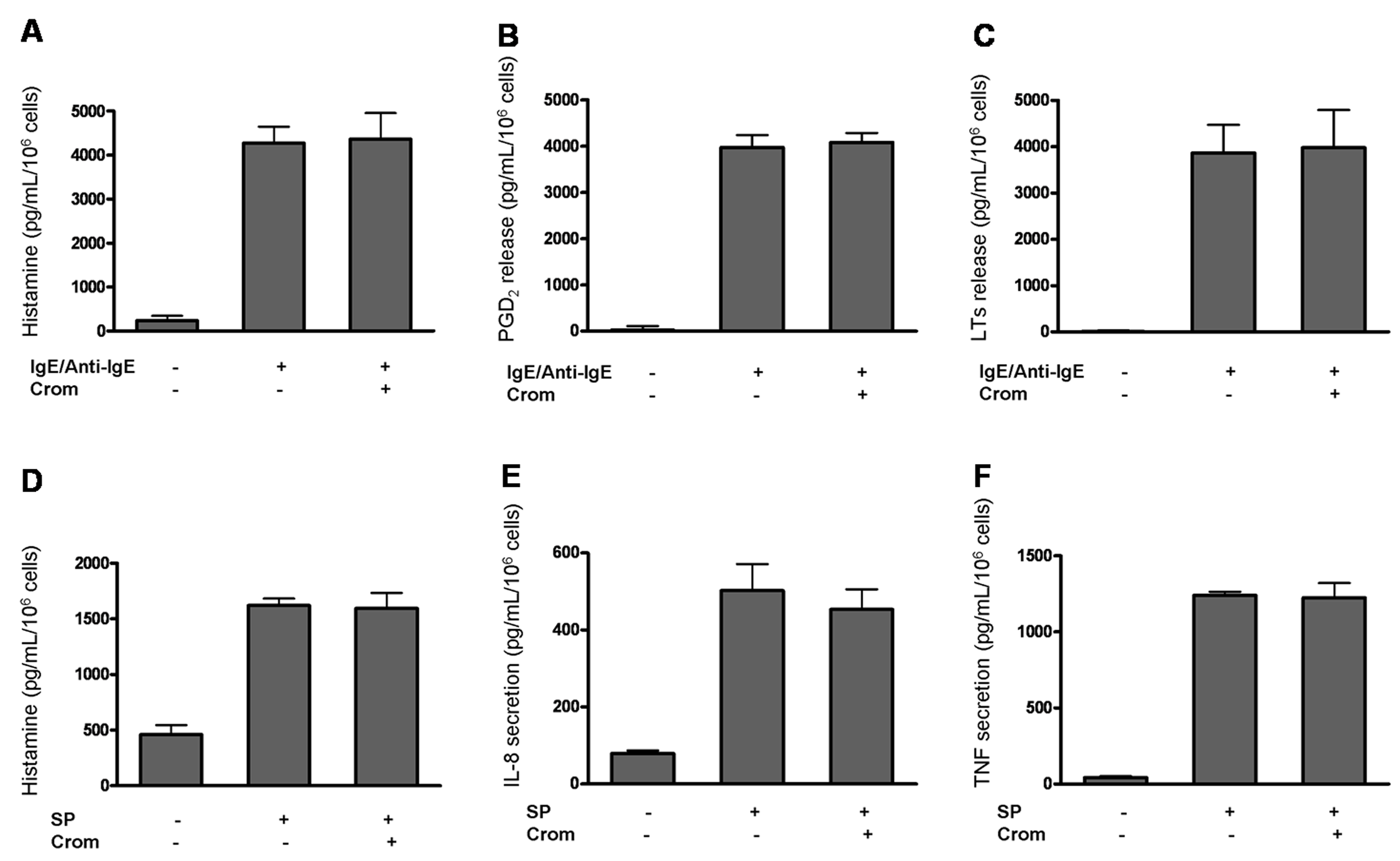

Supplement: Figure S4 — Cromolyn does not show significant inhibition on human mast cells when added 30 min prior to the trigger. Human mast cells were stimulated as described in Fig. 1 & 2. In some experiments, cells were treated with Crom (100 µM) 30 min prior to the trigger. (A–C) hCBMCs; (D–F) LAD2. n = 3. (TIF) [file pone.0033805.s004.tif]
